# RETRACTION: Melittin Inducing the Apoptosis of Renal Tubule Epithelial Cells through Upregulation of Bax/Bcl‐2 Expression and Activation of TNF‐*α* Signaling Pathway

**DOI:** 10.1155/bmri/9876947

**Published:** 2026-04-16

**Authors:** BioMed Research International

RETRACTION: Y. Shu, Y. Yang, Y. Zhao, L. Ma, P. Fu, T. Wei, L. Zhang, “Melittin Inducing the Apoptosis of Renal Tubule Epithelial Cells through Upregulation of Bax/Bcl‐2 Expression and Activation of TNF‐*α* Signaling Pathway,” *BioMed Research International*, 2019, 9450368, https://doi.org/10.1155/2019/9450368

The above article, published online on 29 May 2019 in Wiley Online Library (wileyonlinelibrary.com), has been retracted by agreement between the Handling Editor, Shin‐ichi Yokota; and John Wiley & Sons Ltd. The retraction has been agreed due to error identified with the Figures, originally with Figure 3 on PubPeer [1]. Further investigation has identified further concerns with Figures 1 and 2. A summary of the concerns is as follows:

• In Figure 1b, there are repeated elements between panels C and D
• In Figure 2, there are repeated elements between panel D, and the 8 h panel of Figure 2a in [2]
• In Figure 2, there are repeated elements between panel C, and the 24 h panel of Figure 2a in [2]
• In Figure 3, panel B is duplicated in panel D after 90 degree rotation

After assessment of the concerns and the author’s response, the editorial board no longer have confidence in the results and conclusions presented, and the article is therefore retracted.

The authors disagree with the retraction.
